# Comparative antimicrobial activity of Zataria multiflora essential oil nanoformulations against foodborne pathogens

**DOI:** 10.1038/s41598-025-21984-6

**Published:** 2025-11-03

**Authors:** Saeede Forgi, Mahmoud Osanloo, Fatemeh Norouzi, Mohamad Hosein amushahi, Elham Zarenezhad, Mehran Sayadi, Roghayeh Nejati

**Affiliations:** 1 Student Research Committee, Fasa University of Medical Sciences, Fasa, Iran; 2 Department of Food Safety and Hygiene, School of Health, Fasa University of Medical Sciences, Fasa, Iran; 3 Department of Medical Nanotechnology, School of Advanced Technologies in Medicine, Fasa University of Medical Sciences, Fasa, Iran; 4 Department of Microbiology, School of Medicine, Fasa University of Medical Sciences, Fasa, Iran; 5 Noncommunicable Diseases Research Center, Fasa University of Medical Sciences, Fasa, Iran

**Keywords:** Nanotechnology, Essential oil, Antimicrobial agent, *Zataria multiflora*

## Abstract

Foodborne diseases caused by microbial contamination, as a profound global health challenge, driving the search for natural antimicrobial compound. This study investigated the antimicrobial potential of *Zataria multiflora* essential oil (*Z. multiflora* EO) encapsulated in alginate nanoparticles (Alg-EO), chitosan nanoparticles (Chi-EO), and nanoemulsions (NE-EO) against major foodborne pathogens: *Escherichia coli* (*E. coli*), *Salmonella Typhimurium* (*S. Typhimurium*), *Pseudomonas aeruginosa* (*P. aeruginosa*), and *Staphylococcus aureus* (*S. aureus*). The nanoformulations were prepared using spontaneous emulsification and ionic gelation techniques and subsequently characterized for size, zeta potential, and encapsulation efficiency via dynamic light scattering (DLS) and ATR-FTIR spectroscopy. Finally, the antibacterial activity of the nanoformulations was evaluated via the microdilution method. Results revealed that Chi-EO exhibited the smallest hydrodynamic diameter (129 nm) and highest negative zeta potential (− 37 mV), whereas Alg-EO and NE-EO measured 152 nm (− 28 mV) and 144 nm (24 mV), respectively. Notably, Chi-EO demonstrated strongest antimicrobial efficacy, with the lowest IC50 values against *S. aureus* (321 μg/mL), *S. Typhimurium* (460 μg/mL), *E. coli* (367 μg/mL), and *P. aeruginosa* (504 μg/mL). These findings highlight chitosan nanoparticles serve as an efficient delivery system for *Z. multiflora* EO, offering a natural and potent approach to improving food safety and extending shelf life.

## Introduction

Foodborne diseases impose economic and public health burdens, with millions of cases reported annually^1^. A key contributor to these illnesses is microbial contamination of food, which poses ongoing challenges to both the food industry and regulatory agencies^2^. Among the microorganisms responsible for foodborne diseases, bacterial pathogens such as *E. coli*, *Salmonella spp*., *S. aureus*, and *P. aeruginosa* are particularly notable for their ubiquity, adaptability, and virulence^3^.

*E. coli*, a gram-negative bacterium prevalent in the intestines of humans and animals, primarily contaminates food through fecal contact or cross-contamination during processing^4^. Certain strains, most famously enterohemorrhagic *E. coli,* produce Shiga toxins that can lead to severe complications such as hemolytic uremic syndrome^5^. Similarly, *Salmonella spp.*, another gram-negative bacteria, are estimated to cause 93.8 million cases of gastroenteritis and 155,000 deaths worldwide each year^6^.

On the gram-positive side, *S. aureus* poses a unique hazard by generating heat‑stable enterotoxins that remain active even after the bacteria themselves are eliminated, leading to classic food poisoning symptoms^7^. Moreover, *P. aeruginosa*, an opportunistic gram‑negative pathogen, thrives in food production environments by forming biofilms resistant to common disinfectants, thereby complicating sanitation efforts^8^.

Conventional preservation techniques, including thermal processing, chemical additives, and modified atmosphere packaging, face drawbacks such as sensory changes in foods, consumer reluctance toward synthetic preservatives, and the emergence of resistant microbial strains^9^. These limitations have driven the search for natural, consumer-friendly alternatives, particularly plant-derived essential oils (EOs) with antimicrobial properties.

Owing to their broad-spectrum antimicrobial, antioxidant, and anti-inflammatory properties, plant EOs have garnered attention as potential biopreservatives^10,11^. Among them, *Z. multiflora* Boiss, a thyme-like aromatic herb native to Iran, Pakistan, and Afghanistan, is particularly promising: its phenolic constituents thymol and carvacrol disrupt bacterial membranes, causing leakage of cellular contents and cell death^11,12^.

However, the practical incorporation of EOs in food systems is hampered by their hydrophobicity, volatility, poor water solubility, and strong flavors at effective concentrations^13^. To overcome these challenges, nanotechnology offers innovative delivery platforms that enhance EOs stability and bioavailability. By manipulating materials at the nanoscale (1–200 nm), these systems leverage unique physicochemical properties distinct from bulk materials^14,15^.

In food preservation, such nano-delivery systems include nanoemulsions, polymeric nanoparticles, and encapsulation techniques^16^. Nanoemulsions of kinetically stable dispersions of oil droplets (20–200 nm) stabilized by surfactants provide increased interfacial area, superior stability, and improved bioactive compound uptake^17,18^. Alginate and chitosan are common as encapsulation matrices because of their biocompatibility, biodegradability, and regulatory approval for food applications. Alginate nanoparticles, formed by ionic gelation of seaweed‑derived polysaccharides with calcium ions, yield mucoadhesive carriers that protect encapsulated EOs and enable controlled release^19–21^.

Chitosan nanoparticles, derived from chitin deacetylation, exhibit intrinsic antimicrobial activity by electrostatically binding to and disrupting bacterial cell walls, due to their polycationic nature and high surface to volume ratio. When combined with antibacterial agents such as EOs, chitosan nanoparticles can produce synergistic effects, lowering the minimum inhibitory concentration (MIC) compared to that of free agents^22–24^.

Despite numerous studies on *Z. multiflora* EO and nanotechnology-based delivery systems, comprehensive comparisons of their efficacy among nanoemulsions, alginate, and chitosan nanoparticles against multiple foodborne pathogens remain limited. Therefore, this study compared the antibacterial efficacy of *Z. multiflora* EO loaded nanoemulsions, Alg-EO, and Chi-EO against *E. coli*, *S. Typhimurium*, *S. aureus*, and *P. aeruginosa*, aiming to develop the best natural strategies for enhancing food safety and extending shelf life.

## Materials and methods

### Materials

The *Z. multiflora* EO used in this study was supplied by Barij Essence Pharmaceutical Company (Kashan, Iran). Tween® 20 (polysorbate 20), chitosan (medium molecular weight), sodium tripolyphosphate (TPP), sodium alginate, and calcium chloride dihydrate (CaCl₂·2H₂O) were purchased from Merck (Darmstadt, Germany). The bacterial strains *E. coli* (ATCC 25,922), *S. Typhimurium* (ATCC 13,076), *S. aureus* (ATCC 25,923) and *P. aeruginosa* (ATCC 27,853) utilized in the experiments were obtained from the Persian Type Culture Collection.

### GC‒MS analysis

An Agilent 6890 gas chromatograph equipped with a BPX5-type column (30 m length, 0.25 mm internal diameter, and 0.25 µm film thickness) was used for the analysis. To identify the constituents of the essential oil, a 1 µL sample diluted in n-hexane was injected into the GC/MS system. The temperature program for the column was set as follows: initial oven temperature of 50 °C, held for 5 min, followed by a thermal gradient of 3 °C per minute up to 240 °C, a further increase at a rate of 15 °C per minute to 300 °C, and finally holding at this temperature for 3 min. The total run time was 75 min. The injection port temperature was set to 250 °C in split mode (1:35), and helium was used as the carrier gas, with a flow rate of 0.5 mL/min. The mass spectrometer used was an Agilent 5973 model instrument operating at an ionization voltage of 70 eV in electron ionization (EI) mode with an ion source temperature of 220 °C. The mass range was scanned from 40 to 500 m/z. Data acquisition and analysis were performed using ChemStation software. The identification of the spectra was achieved by comparing their retention indices with those reported in reference books and articles, as well as by matching mass spectra with standard compounds and utilizing data from a computer library^25^.

### Preparation of alginate nanoparticles containing Z. *multiflora* EO

Alginate nanoparticles (AlgNPs) loaded with *Z. multiflora* EO were fabricated via the ionic gelation method. First, 12.5 µL of *Z. multiflora* EO and 10 µL of Tween 20 (serving as a surfactant) were combined and stirred on a magnetic stirrer at 2000 rpm for 3 min at room temperature to achieve a homogeneous mixture. Next, 2.5 mL of a 0.5% (w/v) sodium alginate aqueous solution was slowly added, and the mixture was stirred for an additional 5 min. Finally, a 0.05% (w/v) calcium chloride solution was added dropwise to the mixture while stirring continuously for 40 min to promote ionic crosslinking between the calcium ions and alginate chains, facilitating nanoparticle formation^26^.

### Preparation of chitosan nanoparticles containing Z. *multiflora* EO

Chitosan nanoparticles (ChiNPs) were prepared using a modified ionic gelation technique. First, 0.25% (w/v) chitosan powder was dissolved in a 1% (v/v) acetic acid solution and stirred at 2000 rpm for 4 h at room temperature to obtain a clear chitosan solution. Separately, 12.5 µL of *Z. multiflora* EO and 50 µL of Tween 20 were mixed in a vial and homogenized at 2000 rpm for 3 min. Then, 4.5 mL of the prepared chitosan solution was gradually introduced into the EO mixture under continuous stirring. Subsequently, 400 µL of trisodium phosphate (TPP) solution (1%) was added dropwise. The resulting mixture was stirred at 2000 rpm for 40 min to ensure proper ionic interaction and the formation of ChiNPs^27^.

### Preparation of nanoemulsion containing Z. *multiflora* EO

To prepare the nanoemulsion, 12.5 µL of *Z. multiflora* EO and 40 µL of Tween 20 were placed in a vial and stirred at 2000 rpm for 3 min at room temperature to achieve a uniform dispersion. Subsequently, 5 mL of distilled water (aqueous phase) was slowly added dropwise to the EO-surfactant mixture. The resulting emulsion was then stirred continuously at 2000 rpm for 40 min to complete nanoemulsion formation^28^.

For all the methods used to prepare the nanoformulations (alginate, chitosan, and nanoemulsion), an EO-free form was also prepared in the same manner; however, during the manufacturing process, the *Z. multiflora* EO was omitted. These samples were used as negative controls in antibacterial assessments to compare and evaluate the specific effects of the EO.

### Characterization of the prepared nanoformulations

To analyze the behavior of particles in solution of Alg-EO, Chi-EO, and NE-EO, dynamic light scattering (DLS) was employed via a nanoparticle size analyzer (SZ-100 series, HORIBA Scientific, Japan). Additionally, the droplet size distribution (span) was calculated using the equation (D90—D10) /D50, where D represents the droplet diameter, and the subscripts 90, 10, and 50 denote the diameters below which 90%, 10%, and 50% of the droplets are distributed, respectively. An optimized nanoemulsion is characterized by droplet sizes of < 200 nm and a span value of < 1^29^.

The zeta potentials of the alginate nanoparticles, chitosan nanoparticles, and the selected nanoemulsion were measured using a zeta sizer. Moreover, to determine the morphology of the nanoformulations, a transmission electron microscope (TEM; Philips, Netherlands) was used. A drop of each nanoformulation was placed on a carbon-coated copper grid (200 mesh) and allowed to dry before analysis under the TEM^30^**.**

The chemical properties of *Z. multiflora* EO, Alg-EO, Alg-free, Chi-EO, Chi-free, NE-EO, and NE-free were examined using an ATR-FTIR spectroscope (Bruker, Tensor II, Germany) at room temperature. The analysis was conducted across a wavenumber range of 400–4000 cm^−1^. ATR-FTIR spectroscopy was employed to qualitatively confirm the successful encapsulation of the EO within the nanoformulations.

### Determination of the antibacterial effects of nanoformulations

The antibacterial potential of Alg-EO, Chi-EO and NE-EO against *E. coli*, *S. Typhimurium*, *S. aureus*, and *P. aeruginosa* was investigated via the microdilution method in 96-well microplates. Prior to the test, the bacterial strains were cultured for 18 h on Brain Heart Infusion^13^ agar and incubated at 37 °C. A turbidity equivalent to the 0.5 McFarland standard was prepared for each bacterial suspension, and the optical density was measured using a spectrophotometer (manufactured by UNICO) at a wavelength of 600 nm to standardize the bacterial inoculum. Various dilutions of Alg-EO, Chi-EO and NE-EO were prepared using normal saline, with concentrations ranging from 138 to 2500 ppm. Similarly, dilutions of the EO alone were prepared using phosphate-buffered saline (PBS) at the same concentration range (ppm). In each well of the microplate, 30 µL of the bacterial suspension, 70 µL of BHI broth, and 100 µL of the corresponding nanoformulation or EO dilution were added. For the control group wells, 30 µL of bacterial suspension, 70 µL of BHI broth, and 100 µL of normal saline were inoculated. In the blank group wells, 100 µL of BHI broth and 100 µL of normal saline were added. The microplates were then incubated at 37 °C for 24 h. Following incubation, the optical density of each well was measured using a microplate reader (Synergy). Concurrently, Mueller Hinton agar plates were prepared, and 10 µL from each well of the microplate was streaked onto the agar plates. These plates were then incubated at 37 °C for 24 h.

### Statistical analyses

All the experimental procedures were conducted in triplicate to ensure reproducibility, and the results were expressed as the mean values accompanied by their corresponding standard deviations. The software CalcuSyn (Biosoft, United Kingdom) was used to determine the IC50 values for each sample.

## Results

### GC/MS analysis

GC/MS analysis identified approximately 99% of the compounds in *Z. multiflora* EO. The compounds that were identified are presented in Table 1. Carvacrol (42.35%), thymol (28.95%), and cymene (8.76%) were the major compounds in the *Z. multiflora* EO.

**Table 1:** GC/MS analysis of *Z. multiflora* EO.

No	RT	%	Components	KI	Type
**1**	**11.22**	**1.12**	**alpha.-Pinene**	**939**	**MH**
2	13.54	0.19	beta.-Pinene	979	MH
3	14.11	0.58	beta.-Myrcene	991	MH
4	15.59	0.40	alpha.-Terpinene	1017	MH
**5**	**16.10**	**8.76**	**Cymene**	**1025**	**MH**
6	16.24	0.34	Limonene	1029	MH
**7**	**16.43**	**2.01**	**Eucalyptol**	**1031**	**MO**
**8**	**17.78**	**1.78**	**gamma.-Terpinene**	**1060**	**MH**
**9**	**19.99**	**1.42**	**Linalool**	**1097**	**MO**
10	23.82	0.16	Borneol	1169	MO
11	24.17	0.77	Terpinen-4-ol	1177	MO
12	24.97	0.72	alpha-Terpineol	1188	MO
13	26.43	0.5	Thymol, methyl ether	1235	MO
**14**	**26.85**	**1.05**	**Carvacrol, methyl ether**	**1244**	**MO**
15	29.05	0.22	Bornyl acetate	1285	MO
16	29.40	0.16	Anethole < (E)- >	1284	MO
**17**	**29.63**	**28.95**	**Thymol**	**1290**	**MO**
**18**	**30.05**	**42.35**	**Carvacrol**	**1299**	**MO**
**19**	**31.80**	**1.05**	**Thymol acetate**	**1352**	**MO**
**20**	**32.66**	**1.78**	**Carvacrol acetate**	**1372**	**MO**
**21**	**34.89**	**1.15**	**Caryophyllene E**	**1419**	**SH**
**22**	**35.70**	**1.02**	**Aromadendrene**	**1441**	**SH**
23	37.89	0.46	Viridiflorene	1496	SH
24	41.53	0.71	Spathulenol	1578	SO
**25**	**41.72**	**1.47**	**Caryophyllene oxide**	**1583**	**SO**
		**99.12**	**Total Identified**		

*MH* Monoterpene Hydrocarbons, *MO* Oxygenated Monoterpenes, *SH* Sesquiterpene Hydrocarbons, *SO* Oxygenated Sesquiterpenes.

The values above 1% are indicated in bold.

### Size and distribution of the nanoformulations

**Fig. 1:**
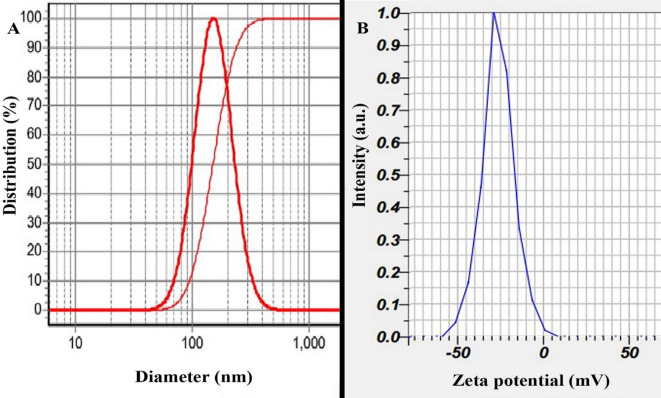
DLS analysis of Alg-EO (**A**) and its zeta potential profile (**B**).

**Fig. 2:**
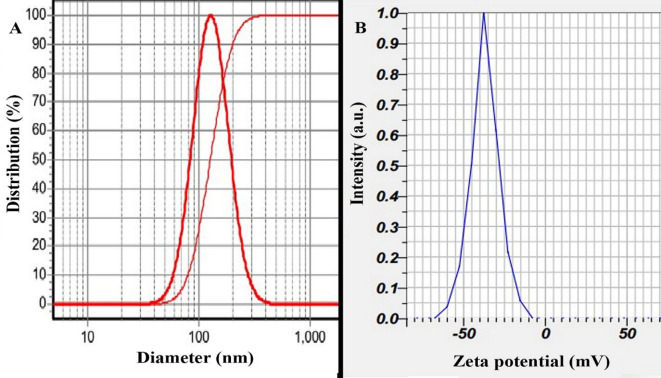
DLS analysis of Chi-EO (**A**) and its zeta potential profile (**B**).

**Fig. 3:**
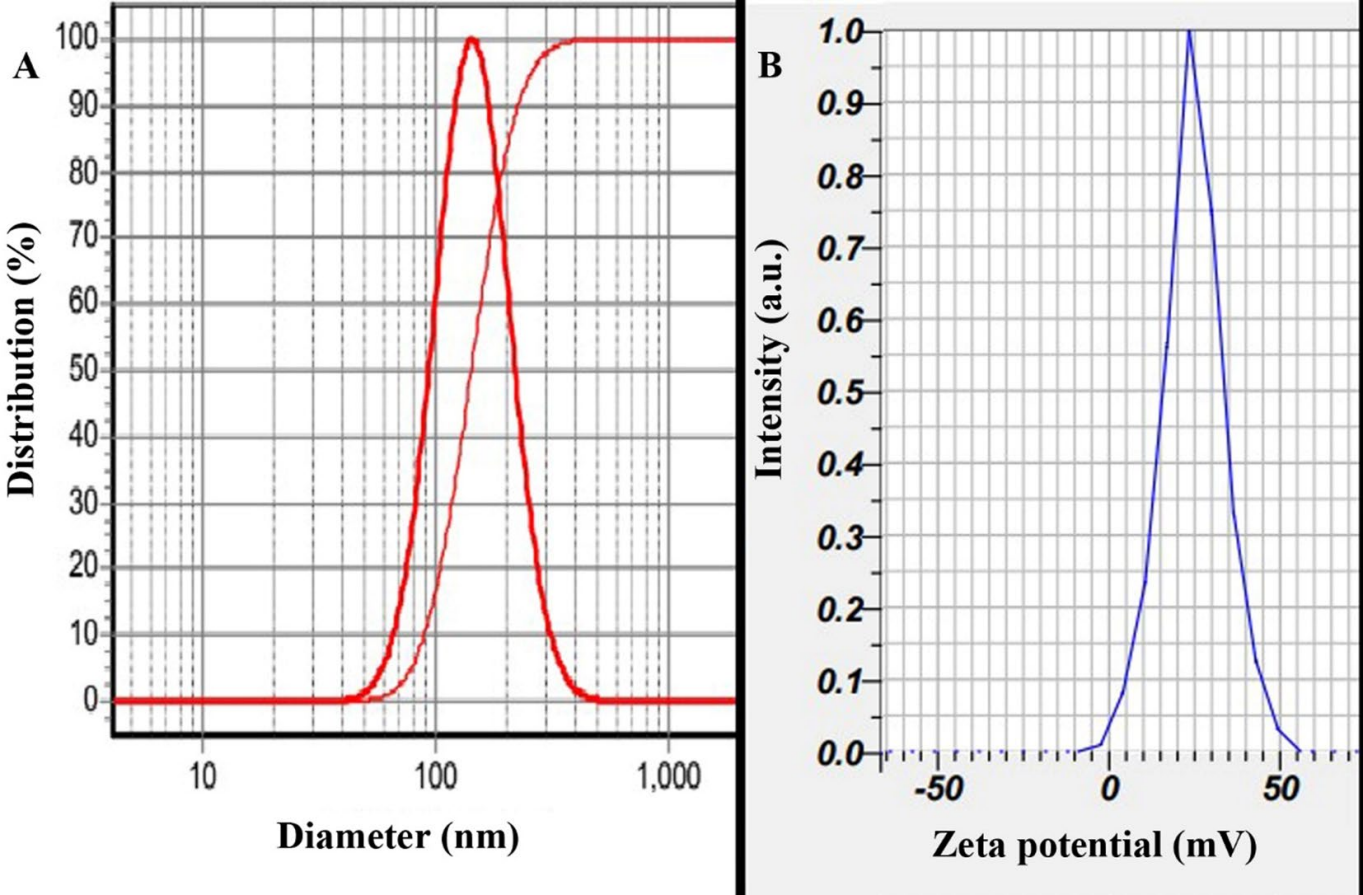
DLS analysis of NE-EO (**A**) and its zeta potential profile (**B**).

**Fig. 4:**
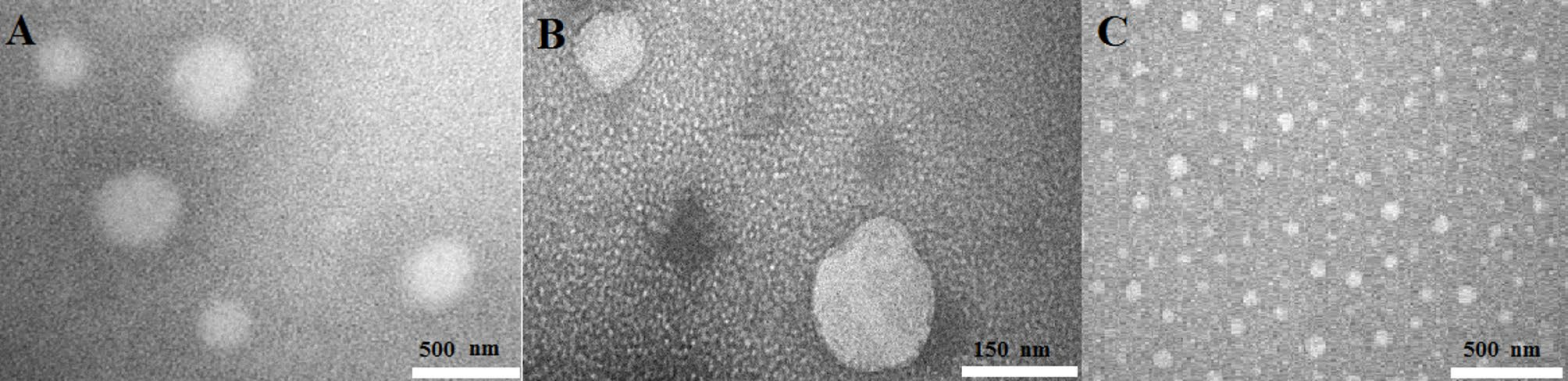
TEM images of (**A**): Alg-EO, (**B**): Chi-EO, and (**C**): NE-EO.

The prepared nanoparticles were characterized using DLS analysis, which revealed that the Alg-EO, Chi-EO, and NE-EO had hydrodynamic diameters of 152 nm, 129 nm, and 144 nm, respectively, with narrow size distributions (SPAN values of 0.97, 0.96, and 0.97), as illustrated in (A) spectrum in Figs. 1, 2 and 3. Furthermore, zeta potential measurements (B in Fig. 1, 2 and 3) demonstrated that the nanoparticles exhibited negative surface charges of − 28 ± 2 mV, − 37 ± 2 mV, and 24 mV for Alg-EO, Chi-EO, and NE-EO, respectively. TEM analysis confirmed spherical nanoparticle morphology in all nanoformulations (Alg-EO, Chi-EO, NE-EO), as shown in Fig. 4.

### Fourier-transform infrared spectroscopy (FTIR)

**Fig. 5:**
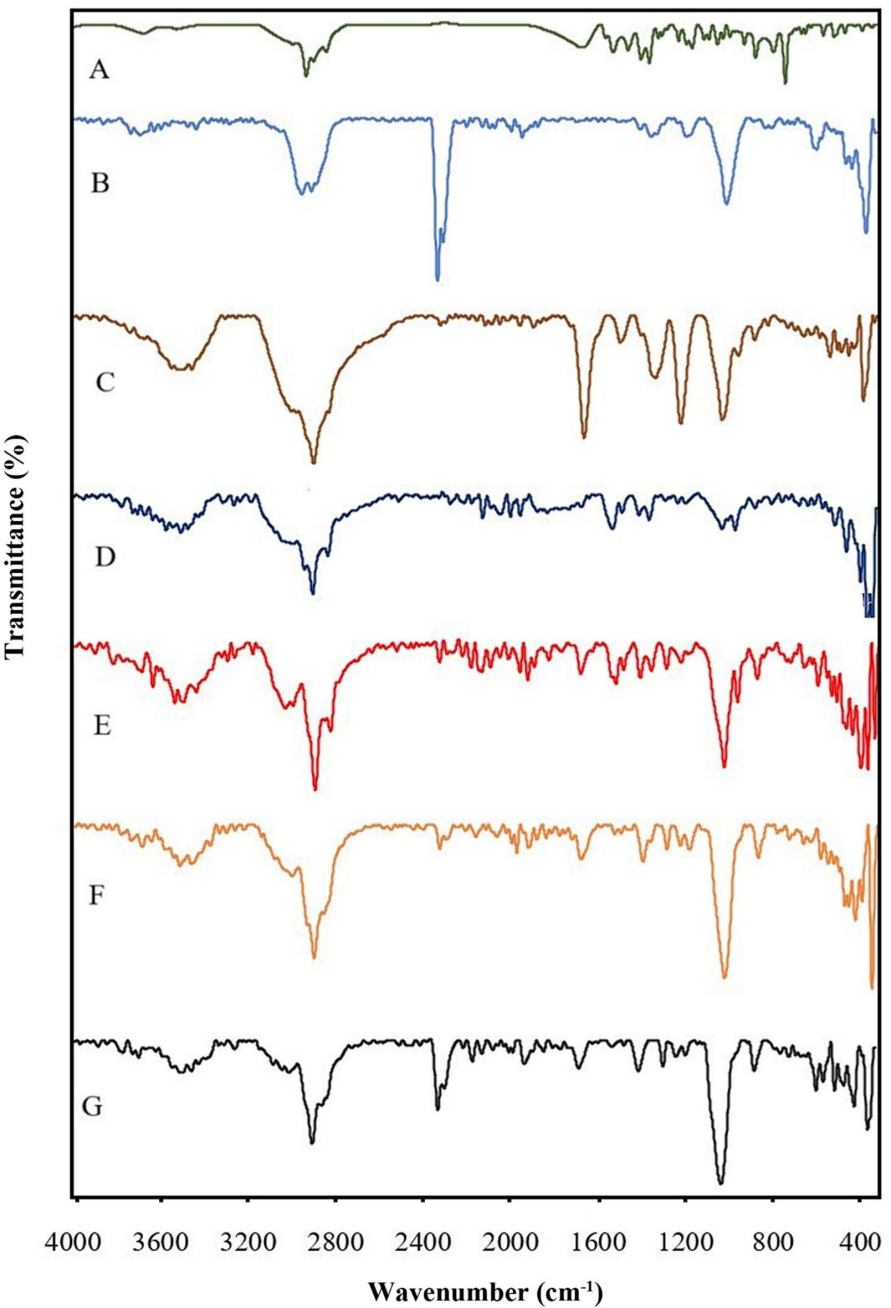
ATR-FTIR spectra of *Z. multiflora* EO (**A**), Chi-EO (**B**), Chi-free(**C**), Alg-EO (**D**), Alg-free (**E**), NE-EO (**F**), NE-free (**G**).

The ATR-FTIR spectrum of *Z. multiflora* EO (A in Fig. 5) revealed that the broad band between 3300 and 3650 cm^−1^ is related to the hydroxyl group resulting from hydrogen bonding between phenolic and alcoholic groups in the essential oil. At 3019 cm^−1^, C=C–H stretching vibrations are displayed; the stretching vibrations at 2959, 2926 and 2870 cm^−1^ are connected to C–H, and the absorption at 1718 cm^−1^ is attributed to the carbonyl group. There are C=C stretching vibrations at 1619 and 1455 cm^−^1. The characteristic band at 1088 cm^−1^ corresponds to C–O vibrations.

The ATR-FTIR Chi-EO (B) spectrum in Fig. 5 showed a broad band at 3200–3700 cm^−1^, indicating hydrogen bonding between the EOs, chitosan, trisodium phosphate and Tween 20. The stretching vibrations at 2924 cm^−1^ are related to C–H arising from the alkane. The stretching vibrations at 2924 cm^−1^ are related to C–H arising from the alkane structure in chitosan and EO. The peak at 1739 cm^−1^ is related to the (C=O) bond. The appearance of a strong new peak at 1250 cm^−1^, attributed to C–N stretching, indicates the formation of a complex through electrostatic interactions between the NH3 + groups of chitosan and the phosphoric groups of TPP (trisodium phosphate) in the nanoparticles. All the characteristic peaks observed in the blank spectrum of the chitosan nanoparticles were also present in the spectrum of the chitosan nanoparticles containing *Z. multiflora* EO at almost the same wave numbers. This result can be attributed to the increase in ionic cross-linking between the –NH3 + groups of the chitosan ions and TPP (trisodium phosphate).

Figure 5C shows the ATR-FTIR spectrum of the Chi-free sample (Chi-EO without *Z. multiflora* EO), and a broad band at approximately 3700–3200 cm^−1^ was found to indicate the hydrogen bonding of OH and N–H stretching vibrations. The absorption at 2925 cm^−1^ is due to the SP3 hybridization in alkanes and C–H stretching. The strong absorption at 1712 cm^−1^ is related to the carbonyl group in tween 20. The strong band at 1279 cm^−1^ can be attributed to the C–N stretching due to the interpenetration between chitosan and trisodium phosphate. The strong bands at 1093 cm^−1^ are related to the symmetric and antisymmetric stretching vibrations in the PO2 group. In addition, the strong bands at 1012 cm^−1^ are attributed to the symmetric and antisymmetric stretching vibrations in the PO3 group.

In Fig. 5D, the ATR-FTIR spectrum of Alg-EO shows a broad band between 3200 and 3700 cm^−1^, which is attributed to the hydroxyl groups involved in hydrogen bonding. The vibrations at approximately 2957 and 2852 cm^−1^ were related to C–H stretching vibrations. A band confirmed the presence of carbonyl groups in *Z. multiflora* EO and Tween 20. The bands at 1583 and 1347 cm^−1^ were related to the symmetric and asymmetric stretching vibrations of carbonyl groups. The characteristic band at 1095 cm^−1^ is related to the reaction between carboxyl groups and calcium ions (formation of CO–Ca–CO group structures) and indicates an increase in C–O vibrations. This peak confirmed the occurrence of ionic crosslinking. Furthermore, physical cross-linking between the hydroxyl groups of alginate, 20, and EO resulted in the consumption of a small number of hydroxyl groups.

The ATR-FTIR spectrum of Alg-free (Alg-EO without *Z. multiflora* EO) in Fig. 5E shows a broad band between 3200 and 3700 cm^−1^, which is related to the hydroxyl groups in Tween 20, water and alginate. The peak at approximately 3000 cm^−1^ is related to C=C–H, and the stretching vibration around 2900 cm^−1^ is related to C–H. Additionally, a carbonyl group was observed at 1734 cm^−1^ in Tween 20. Due to the presence of sodium alginate, the symmetric and asymmetric stretching vibrations of the carbonyl group were observed at 1577 cm^−1^ and 1418 cm^−1^, respectively. The bands at 1093 cm^−1^ are attributed to the stretching vibrations of the C–O group.

The ATR-FTIR spectrum of NE-EO in Fig. 5F, showed a broad band around 3200–3700 cm^−1^, which was assigned to the stretching vibration of OH groups due to hydrogen bonding in *Z. multiflora* Boiss. The stretching vibration at 2952 cm^−1^ was attributed to C–H in the EO and tween 20. The absorption peak at 1735 cm^–1^ confirmed the presence of a carbonyl group in the EO and the tween 20. The strong band at 1094 cm^−1^ could be attributed to CO vibrations. The observed shift of the peaks to lower wave numbers confirmed the increase in hydrogen bonding among the EOs, Tween 20 and water within the NE.

As shown in Fig. 5G, the ATR-FTIR spectrum of the NE-free sample (NE-EO without *Z. multiflora* EO) presented a broad peak between 3300 and 3700 cm^−1^, which is characteristic of the stretching vibrations of the hydroxyl group in Tween 20 and water. The peaks at 3098 and 3191 cm^−1^ are related to the C–H stretching vibrations. In addition, the stretching vibrations at 2923 and 2881 cm^−1^ can be attributed to the C–H in Tween 20. The band at 1729 cm^−1^ was associated with the stretching vibration of the C=O group in Tween 20. Finally, the strong peak at 1092 cm^−1^ can be related to the C–O stretching vibrations.

### Antibacterial effects of the nanoformulations

**Fig. 6:**
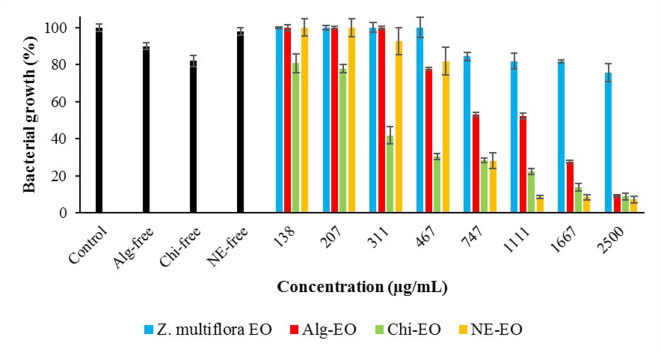
Antibacterial effects of nanofomulations against *E. coli.*

**Fig. 7:**
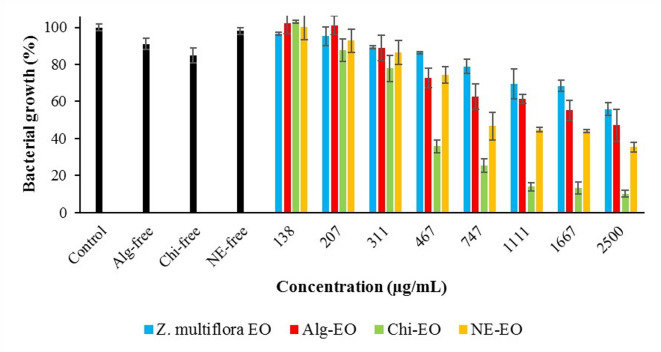
Antibacterial effects of nanofomulations against *P. aeruginosa.*

**Fig. 8:**
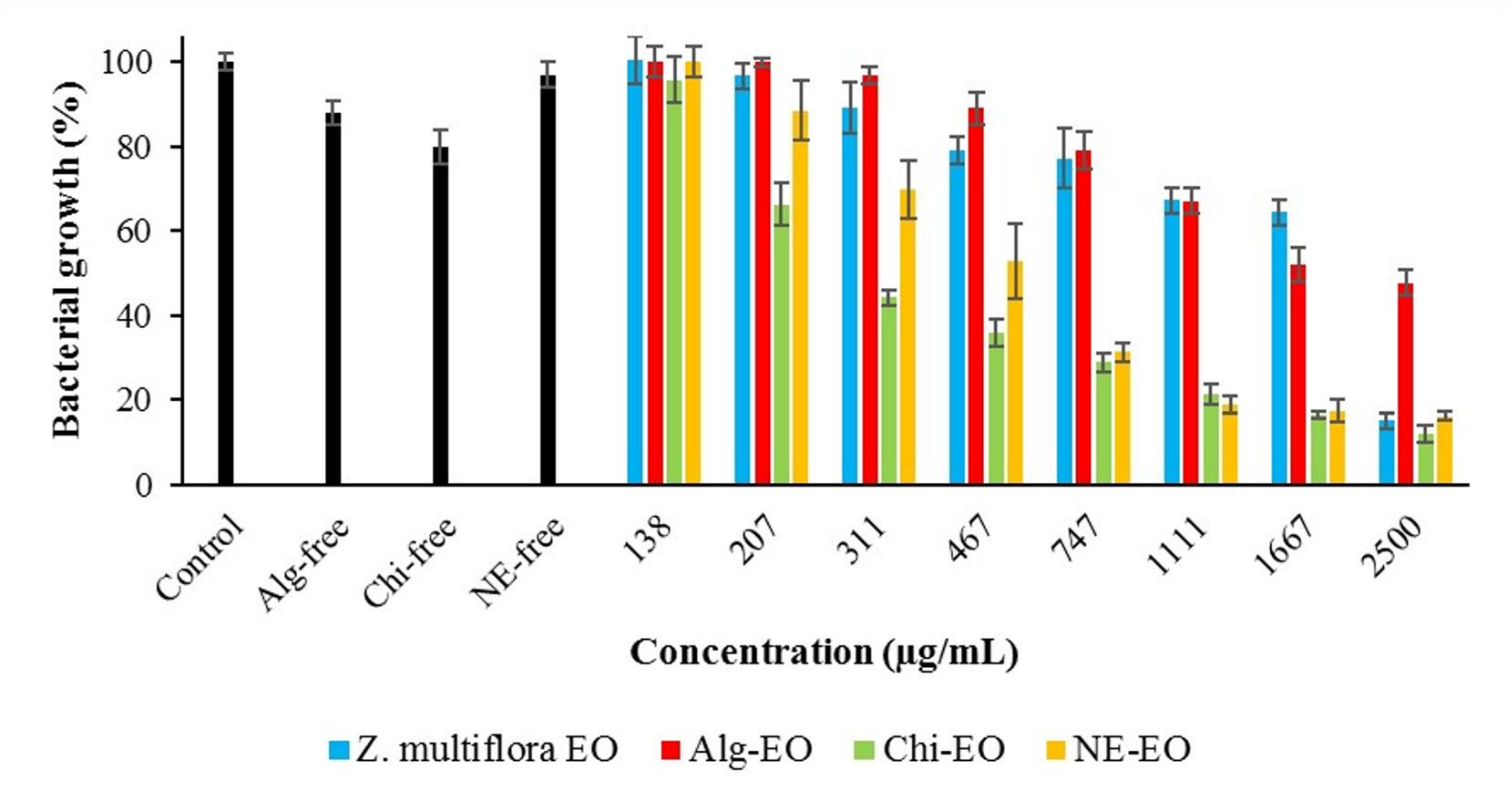
Antibacterial effects of nanofomulations against *S. Typhimurium.*

**Fig. 9:**
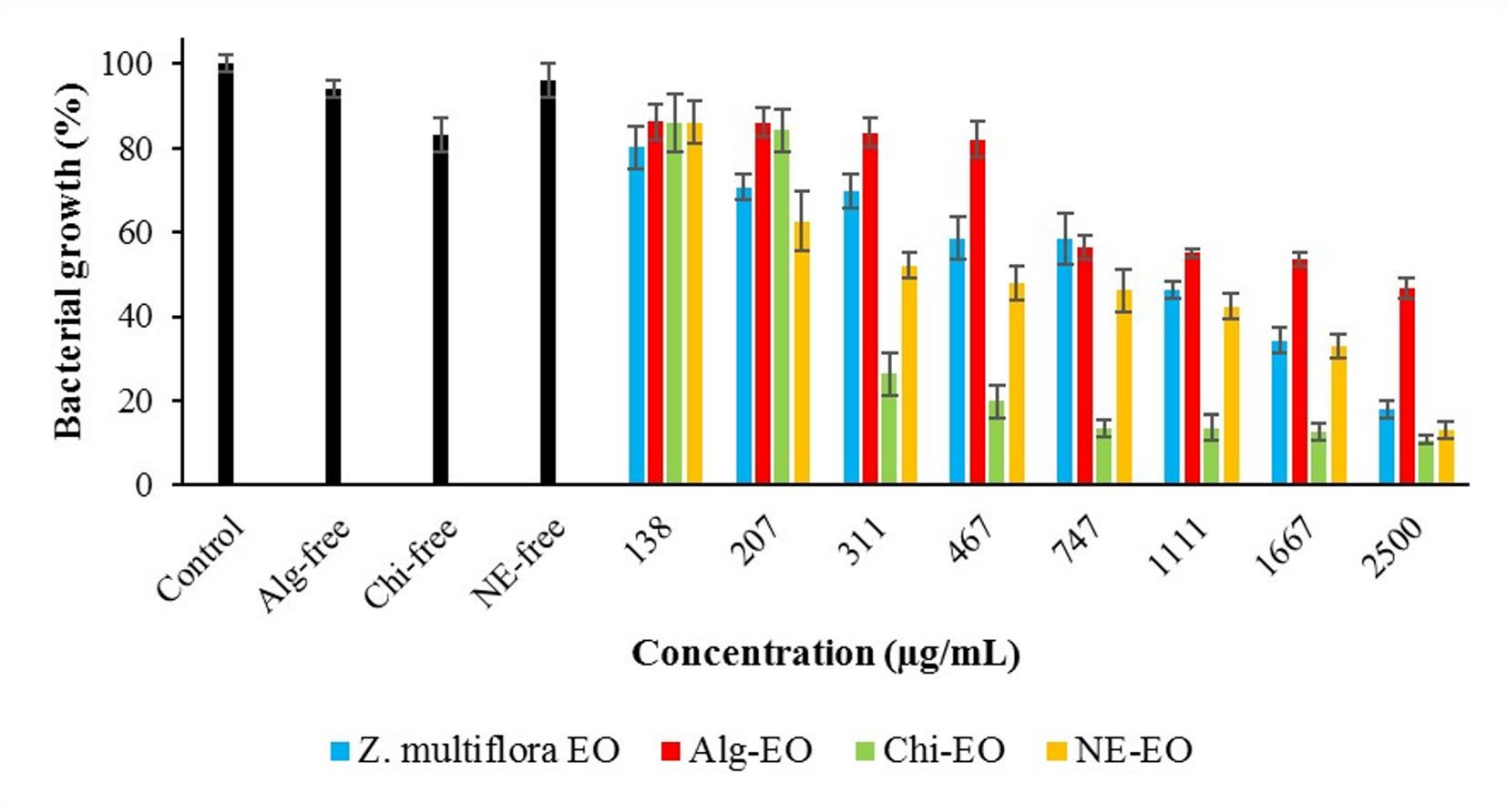
Antibacterial effects of nanofomulations against *S. aureus.*

Results of antibacterial effects of nonformulated *Z. multiflora* EO and the prepared nanoformulations Alg-EO, Chi-EO, and NE-EO against *E. coli, P. aeruginosa, S. Typhimurium*, and *S. aureus* are shown in Figs. 6–9. The bacterial growth inhibition data revealed that Chi-EO exhibited the strongest antimicrobial activity, reducing growth to < 10% for *E. coli* (8.8%) and *P. aeruginosa* (10.2%) at the highest concentration (2500 μg/mL), while NE-EO nearly completely suppressed *E. coli* (7.2%). In contrast, Alg-EO showed limited efficacy, inhibiting growth by only 9.3% for *E. coli* and 47.6% for *S. Typhimurium* at 2500 μg/mL. Essential oil-loaded nanoformulations (Alg-EO, Chi-EO, and NE-EO) significantly outperformed nanoparticle-only controls (Alg-free, Chi-free, and NE-free), which exhibited minimal intrinsic antibacterial activity (82–98% growth).

Furthermore, the obtained IC50 values of these compounds against the abovementioned pathogenic bacteria are summarized in Table 2. Chi-EO exhibited the strongest antibacterial activity, consistently showing the lowest IC50 values across all tested strains (367 μg/mL for *E. coli*, 504 μg/mL for *P. aeruginosa*, 460 μg/mL for *S. Typhimurium*, and 321 μg/mL for *S. aureus*), with narrow confidence intervals (LCL-UCL), indicating high statistical reliability. In contrast, NE-EO demonstrated moderate efficacy, outperforming pure essential oil but remaining less effective than Chi-EO. Alg-EO showed the weakest activity, even underperforming the unmodified essential oil against *E. coli* (IC50=917 μg/mL) and *S. aureus* (IC50=1854 μg/mL). Notably, the pure essential oil lacked measurable inhibitory effects against *E. coli* (NC*), while all the nanoformulations restored activity, highlighting their role in enhancing bioavailability.

**Table 2:** Obtained IC50 values (μg/mL) of Ag-EO, Chi-EO, and NE-EO.

Factors	*Z. multiflora* EO			Alg-EO		Chi-EO		NE-EO	
		*E. coli*							
IC50		NC*		917		367		684	
LCL	UCL	NC*	NC*	768	1095	289	465	521	898
		*P. aeruginosa*							
IC50		2868		1891		504		1168	
LCL	UCL	2277	3613	1260	2837	385	661	879	1552
		*S. Typhimurium*							
IC50		1423		1893		460		571	
LCL	UCL	987	2054	1477	2426	317	665	453	719
		*S. aureus*							
IC50		724		1854		321		534	
LCL	UCL	582	901	1223	2810	191	539	387	738

*Not calculated due to lower efficacy than 50%

The antibacterial efficacy varied by bacterial strain. For gram-negative bacteria, Chi-EO reduced the IC50 of Ag-EO by fivefold against *P. aeruginosa* (2868 vs. 504 μg/mL) and threefold against *S. Typhimurium* (1423 vs. 460 μg/mL). Chi-EO achieved an IC50 of 321 μg/mL against gram-positive *S. aureus*, which was significantly lower than that of Ag-EO (724 μg/mL). NE-EO also improved the activity compared with that of the pure oil but was less potent than Chi-EO. However, the performance of Alg-EO was inconsistent, with higher IC50 values than those of Ag-EO in some cases (e.g., *S. aureus*), suggesting potential formulation-related limitations.

## Discussion

Food has long been recognized as a favorable medium for microbial proliferation, often leading to notable outbreaks of foodborne diseases^31^. Effective, natural methods of food preservation are currently gaining momentum, as consumers increasingly prefer minimally processed foods without artificial additives^32^.

In the present survey, the antimicrobial efficacy of *Z. multiflora* EO and three nanoformulations containing *Z. multiflora* EO, Alg‑EO, Chi‑EO, and NE‑EO against four major foodborne pathogens, *E. coli*, *P. aeruginosa*, *S. Typhimurium*, and *S. aureus,* was evaluated.

In this study, GC–MS analysis of *Z. multiflora* EO revealed that carvacrol (42.35%) and thymol (28.95%) constitute the major bioactive components, consistent with prior characterizations of Iranian-origin *Z. multiflora* EO^33^. Although some variations are well-documented, they may stem from differences in geographical location, harvest season, and environmental conditions^34^.

The observed antimicrobial effect can be attributed to the high concentration of phenolic compounds (71.3% combined carvacrol and thymol), which disrupt microbial membranes through hydrophobic interactions and protonophore activity 35. Notably, the carvacrol-to-thymol ratio of 1.46 falls within the reported optimal range for synergistic antimicrobial effects 36, suggesting this specific chemical profile may be particularly effective against foodborne pathogens.

The FTIR of *Z. multiflora* EO into chitosan biopolymer nanoparticles confirmed that the present of Chitosan’s characteristic peaks at 3265 cm⁻^1^ related to N–H stretching and the band at 1650 cm⁻^1^ showed C=O stretching of amide I in chitosan, also the characteristic band at 2920 cm⁻^1^ demonstrated the present of C–H stretching of alkanes and the spectra at 1740 cm⁻^1^ related to carbonyl group in* Z. multiflora* EO^37^.

According to previous research, hydrogen bonding and ionic interactions of Alginate nanoparticles containing *Z. multiflora* EO showed the broad bond at 3246 cm⁻^1^ related to hydroxyl group (-OH) and the band at 1596 cm⁻^1^ attributed to carboxylate (–COO⁻) peaks, suggesting hydrogen bonding and calcium ion (Ca^2^⁺) coordination this peak indicating intermolecular hydrogen bonding between EO and alginate^38,39^. Researcher reported that the ATR-FTIR spectrum of nanoemulsions containing *Z. multiflora* EO showed the broad band between 3200 and 3500 cm⁻^1^ related to (OH/N–H) group due to hydrogen bonding^40^.

DLS analysis revealed that Chi‑EO exhibited the smallest particle size (129 nm), followed by NE‑EO (144 nm) and Alg‑EO (152 nm). These size differences can be attributed to the differing formation mechanisms of each nanoformulation. The smaller size of Chi‑EO is consistent with previous reports using ionic gelation techniques^41^, while the larger particle size of Alg‑EO reflects the calcium-induced crosslinking gelation nature of alginate^42^. Notably, all formulations maintained nanoscale dimensions (< 200 nm), which is crucial for enhanced bioavailability and homogeneous dispersion in food matrices^43^.

Zeta potential stands as a critical determinant of nanoformulation stability. Extensive studies have established that nanoformulations achieve optimal colloidal stability when exhibiting absolute zeta potential values exceeding 30 mV, irrespective of charge polarity^44^. These experimental data demonstrated that Chi‑EO   (− 37.2 mV) displayed enhanced stability compared to other nanoformulations.

These findings demonstrate that Chi-EO exhibited the strongest antimicrobial activity against all the tested pathogens, with significantly lower IC₅₀ values than Alg-EO and NE-EO. The superior performance of Chi‑EO likely stems from the inherent cationic nature of chitosan, which enhances electrostatic interactions with negatively charged bacterial cell envelopes and is particularly effective against gram‑positive *S. aureus*^45,46^. This interaction disrupts membrane integrity, facilitating more efficient delivery of EO components into bacterial cells^46,47^. Moreover, these nanoformulations overcome the key limitations of pure EO hydrophobicity and volatility by stabilizing bioactive compounds and enabling controlled release^48^.

While NE-EO improved EO dispersibility and bioavailability, its less targeted delivery resulted in lower efficacy than Chi-EO. In contrast, the relatively weak activity of Alg‑EO may reflect suboptimal release kinetics or electrostatic repulsion between the negatively charged alginate matrix and bacterial surfaces^49,50^. Notably, control experiments confirmed that the antimicrobial effect was driven by the EO itself: nanoparticle-only controls (e.g., Chi-free: 82% growth for *E. coli* vs. Chi-EO: 8.8%) presented minimal activity^51,52^. Thus, chitosan acts primarily as an adjuvant, enhancing the solubility and cellular uptake of EO compounds rather than serving as the main bactericidal agent.

Compared with prior studies, these results align with findings such as MICs of 290 μg/mL against *S. aureus* and 580 μg/mL against *E. coli* when chitosan thyme EO nanoparticles were used^53^ and MICs of 250 μg/mL against *S. aureus* when chitosan cinnamon EO formulations were used^54^. However, this study revealed some reprived efficacy against *P. aeruginosa* and *S. aureus*^13^, likely due to the high thymol and carvacrol contents of *Z. multiflora* EO^55^.

Mechanistically, chitosan nanoparticles leverage their high surface to volume ratio to increase bacterial contact^56^ and exploit their cationic charge to induce membrane permeability changes, osmotic imbalance, and cell lysis^22,57^. The major EO phenolics carvacrol (42.35%), thymol (28.95%), and cymene (8.76%) further disrupt membranes, inhibit ATPase activity, and generate oxidative stress, culminating in bacterial death^58–62^.

Differences in gram-positive versus gram-negative susceptibility reflect the cell wall architecture: an additional outer membrane in gram-negative bacteria impedes hydrophobic EO penetration. However, the Chi-EO’s mechanism appears to overcome this barrier more effectively than Alg-EO or NE-EO^63,64^. This study has several limitations, including its in vitro focus and lack of sensory evaluation in food matrices. Future work should validate these findings in real food systems and assess their sensory impacts.

## Conclusion

This study demonstrated that, compared with alginate nanoparticles and nanoemulsions, chitosan-based nanoformulations of *Z. multiflora* essential oil significantly enhance antimicrobial efficacy against both gram-positive and gram-negative foodborne pathogens. Chi-EO’s cationic properties and high surface area facilitate superior membrane interaction and controlled EO release, overcoming solubility and volatility issues. These findings highlight Chi-EO as a promising natural preservative strategy for improving food safety and shelf life, warranting further in vivo validation and mechanistic exploration.

## Data Availability

All the data generated or analyzed during this study are included in this published article.
